# Right or Left: The Role of Nanoparticles in Pulmonary Diseases

**DOI:** 10.3390/ijms151017577

**Published:** 2014-09-29

**Authors:** Xuefei Lu, Tao Zhu, Chunying Chen, Ying Liu

**Affiliations:** 1 CAS Key Laboratory for Biomedical Effects of Nanomaterials and Nanosafety, National Center for Nanoscience and Technology of China, Beijing 100190, China; E-Mails: fxlv@mail.ustc.edu.cn (X.L.); chenchy@nanoctr.cn (C.C.); 2 Hefei National Laboratory for Physical Sciences at Microscale and School of Life Sciences, University of Science and Technology of China, Hefei 230027, Anhui, China; E-Mail: zhut@ustc.edu.cn

**Keywords:** nanoparticle, pulmonary pathobiological processes, lung disease, therapy

## Abstract

Due to the rapid development of the nanotechnology industry in the last decade, nanoparticles (NPs) are omnipresent in our everyday life today. Many nanomaterials have been engineered for medical purposes. These purposes include therapy for pulmonary diseases. On other hand, people are endeavoring to develop nanomaterials for improvement or replacement of traditional therapies. On the other hand, nanoparticles, as foreign material in human bodies, are reported to have potential adverse effects on the lung, including oxidase stress, inflammation, fibrosis and genotoxicity. Further, these damages could induce pulmonary diseases and even injuries in other tissues. It seems that nanoparticles may exert two-sided effects. Toxic effects of nanomaterials should be considered when their use is developed for therapies. Hence this review will attempt to summarize the two-side roles of nanoparticles in both therapies for pulmonary diseases and initiation of lung diseases and even secondary diseases caused by lung injuries. Determinants of these effects such as physicochemical properties of nanoparticles will also be discussed.

## 1. Introduction

The rapid development of nanotechnology has brought breakthrough applications in many industrial areas such as electronics [1], biosensors [2], drug delivery [3], DNA Vaccine Adjuvants [4] and so on. Manufactured nanomaterials have even been transformed into various consumer products in our daily life [5], such as carbon-based nanomaterials (fullerene, carbon nanotubes), metal-based nanomaterials (metal oxides, quantum dots), and dendrimers (branched nanosized polymers with a high potential for medical applications) and so on. Additionally, some nanomaterials are produced naturally or unintentionally and spread into the air, soil or water, such as volcanic eruption, marine pollution, and micrometric airborne pollutant particles [6,7].

Nanoparticles or nanomaterials can enter the human body by different routes: inhalation (respiratory tract), ingestion [gastrointestinal (GI) tract], dermal contact (skin), injection (blood circulation) and so on [8]. They may play a positive role, such as the application as drug carriers. On the other hand, they may have adverse effects on the body, such as fibrosis, inflammation or genotoxicity. The safety evaluation of nanomaterials is gradually a popular topic in recent years.

In this review, application in lung diseases therapy and side effects in pulmonary functions of nanoparticles were respectively introduced. We also summarize the penetration, deposition, translocation, and elimination of nanoparticles, and sum up the types and physicochemical characteristics of inhaled nanoparticles. In addition, probably diseases in other systems caused by pulmonary injury also deserve our attention.

## 2. Nanoparticle Therapy in Lung Diseases

Inhalation through the lung is the primary natural pathway. The lung consists of two different parts, airways (transporting the air in and out the lungs) and alveoli (gas exchange areas). The airways are a relatively hard barrier for particles to penetrate through, an active epithelium protected with a viscous layer of mucus, while the barrier between the alveolar wall and the capillaries is very thin and relatively weak in the gas exchange areas [9]. The large surface area of the alveoli and the intense air-blood contact makes the alveoli less well protected against environmental damages. These damages may cause some pulmonary diseases including lung cancer [10]. Additionally, lung is one of the most common target organs where tumor cells tend to migrate in cancer patients. However, many traditional drugs or therapies fail to treat these diseases completely or alleviate symptoms effectively. There are many nanoparticles currently being developed for respiratory applications that aim at overcoming the limitations of conventional drugs (Table 1). Nanoparticles help the treatment of many lung diseases, such as asthma, tuberculosis, emphysema, cystic fibrosis, and cancer.

**Table 1:** Nanoparticles used for respiratory applications.

Nanoparticle	Animal Models	Exposure Method	Description	Use	Ref.
poly(l-aspartic acid-co-lactic acid)/DPPE co-polymer NPs	Mouse xenograft model	intraperitoneal injection	amphiphilic biodegradable poly(l-aspartic acid-co-lactic acid)/DPPE co-polymer NPs loaded with doxorubicin (DOX)	Lung cancer	[3]
PEG-dendritic block telodendrimer	OVA-exposed mice	intravenous injection	self-assembling nanoparticles containing Dex	Allergic Asthma	[11]
pDNA nanoparticles (NPs)	OVA-exposed mice	intranasal	chitosan/IFN-gamma pDNA NPs (CIN)	Allergic Asthma	[12]
poly (dl-lactideco-glycolide) NPs	*M. tuberculosis* infected guinea pigs	inhalation	poly (dl-lactideco-glycolide) loaded with ATDs	Tuberculosis	[13]
polybutyl cyanoacrylate NPs	Mouse xenograft model	intravenous injection	DOX-loaded NPs were incorporated into inhalable effervescent and non-effervescent carrier particles using a spray-freeze drying technique	Lung cancer	[14]
poly(beta-amino ester) (PBAE) polymers	Mouse xenograft model	intratumoral injection	biodegradable PBAE polymers that self-assemble with DNA	Lung cancer	[15]
LPH (liposome-polycation-hyaluronic acid) nanoparticles	Mouse xenograft model	intravenous injection	LPH nanoparticle formulation modified with tumor-targeting single-chain antibody fragment for systemic delivery of siRNA and microRNA efficiently downregulated the target genes (c-Myc/MDM2/VEGF)	Cancer lung metastasis	[16]

### 2.1. Asthma

Asthma, a major public health problem, is believed to be a chronic inflammatory disorder associated with airway hyper-responsiveness. Most of the patients suffered from rhinitis and dyspnea. The chronic inflammation in asthma can lead to ultra-structural changes in airways associated with airway remodeling [17]. The damages are not completely reversed by current available therapeutic strategies such as inhaling steroids. Inhaled steroids are the treatment of choice to control asthma, but their pharmacological effect tends to be short. In addition, their use has been limited due to systemic side effects such as adrenocortical suppression, Cushing’s syndrome and osteoporosis [18].

**Figure 1:**
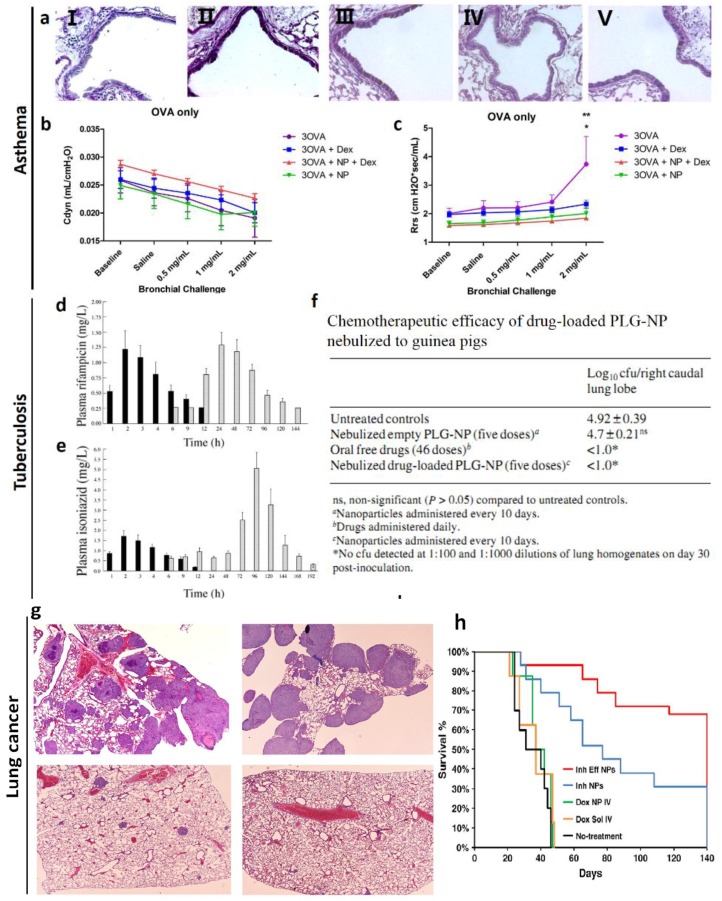
(**a**) Periodic Acid-Schiff staining for goblet cells from representative sections of lobar bronchi or daughter generation airway in mice from air-exposed (I), OVA-exposed (II), OVA-exposed Dex-treated (III), OVA-exposed Dex NP (IV), and OVA-exposed NP (V) treated mice [11]; (**b**) Total lung compliance in mice exposed to either filtered air or 2 weeks of OVA alone [11]; (**c**) Total respiratory system resistance in Balb/c mice exposed to either filtered air or 1 week of OVA alone (treatment with either Dex or itsnanoparticle drug vehicle (NP) independently attenuated Rrs and AHR (*, ** *p* < 0.0001) down to air control levels at the highest dose of methacholine) [11]; (**d**) Plasma profile of following the nebulization of drug-loaded PLG-NP, and oral administration of rifampicin [13]; (**e**) Plasma profile of following the nebulization of drug-loaded PLG-NP, and oral administration of isoniazid [13]; (**f**) Chemotherapeutic efficacy of drug-loaded PLG-NP nebulized to guinea pigs [13]; (**g**) Lung section of mouse from the non-treatment group (I), treated with doxorubicin solution intravenously (II), non-effervescent doxorubicin nanoparticle powder (III) and effervescent doxorubicin nanoparticle powder (IV) [14]; (**h**) Percent animal survival *versus* time [14].

A recent study has shown that dexamethasone contained in self-assembling nanoparticles (Dex-NP) and delivered systemically would target the lung and decrease allergic lung inflammation and airways hyper-responsiveness to a greater degree than equivalent doses of dexamethasone (Dex) alone in asthmatic mice [11] (Figure 1a–c). Mice were sensitized with ovalbumin (OVA) on days 0 and 14 by intraperitoneal injections. And then OVA aerosol exposures began on day 28. Mice were exposed for 30 min, three times per week for the duration of the experiment to induce asthma. Authors found that OVA-exposed mice treated with Dex-NP had significantly fewer total cells and eosinophils in the lung lavage than OVA-exposed mice alone. Also, lower levels of the inflammatory cytokines interleukin (IL)-4 and monocyte chemotactic protein-1 (MCP-1) were found in lungs of the Dex-NP compared to control, and they were not lower in the Dex alone group [11]. In addition, respiratory system resistance was lower in the Dex-NP compared to the other OVA-exposed groups suggesting a better therapeutic effect on airways hyper-responsiveness [11].

In addition, chitosan in the form of nanoparticles (100–200 nm) could be used to deliver plasmids [12]. Kumar *et al.* demonstrated that chitosan interferon (IFN)-γ-plasmid deoxyribonucleic acid (pDNA) nanoparticle therapy effectively exhibited significantly lower AHR to methacholine challenge and less lung histopathology in OVA-sensitized mice [12].

### 2.2. Tuberculosis

Tuberculosis (TB) is a chronic communicable disease caused by the bacterium *Mycobacterium tuberculosis*. Despite of available treatments for tuberculosis for almost half a century, this disease still leads to preventable deaths in the world annually [19]. Nanoparticle-based drug delivery systems may be considered for the treatment of tuberculosis. For example, a single inhalation of poly (dl-lactideco-glycolide) (PLG) nanoparticles loaded with anti-tubercular drugs (ATDs) to guinea pigs resulted in sustained therapeutic drug levels in the plasma for 6–8 days and in the lungs for up to 11 days [13] (Figure 1d–f). *M. tuberculosis* infected guinea pigs were exposed to nebulizated nanoparticles containing drugs every 10th day, no tubercle bacilli could be detected in the lung after five doses of treatment whereas 46 daily doses of orally administered drug were required to obtain an equivalent therapeutic benefit [13]. This study showed that nebulization of nanoparticle-based ATDs improved drug bioavailability and reduced the dosing frequency for better management of pulmonary tuberculosis [13].

### 2.3. Lung Cancer

Lung cancer is one of the most lethal cancers in both men and women [20]. Chemotherapy is primarily an adjuvant to surgery. However, chemotherapeutic agents have apparently adverse effects [21,22].

Many nanoparticle-based delivery systems are designed to accurately carry chemotherapeutic drugs to tumor cells and hence limit their toxicity. For example, tumor bearing Balb/c mice treated with inhalable nanoparticle powder containing 30 μg doxorubicin showed a highly significant improvement in survival compared to all other treatment groups [14]. Pathological samples showed large tumor masses in the lungs of animals not treated or treated with i.v. injections of doxorubicin solution (Figure 1g,h). The lungs of animals treated with inhalable effervescent doxorubicin NPs showed fewer and much smaller tumors compared to the control groups. This work demonstrates inhalable effervescent doxorubicin NPs may be an effective way to treat human lung cancer [14].

In addition to chemotherapeutic drugs, researchers are developing nanoparticle-based delivery systems to miRNAs or siRNAs that silence special oncogene expression or DNA sequence of tumor-suppressor genes to kill cancer cells specally and efficiently [15,16]. In a study, nanoparticle delivery of tumor protein p53 (TP53) gene, which acts as a tumor suppressor and regulates cell division, resulted in expression of exogenous tumor protein p53, induction of apoptosis, and accumulation of cells in sub-G1 [15]. H446 cells, a kind of human small cell lung cancer cell lines, were subcutaneously injected into mice. Intratumoral injection of H446 xenografts with polymers carrying TP53 caused marked tumor growth inhibition. This is the first demonstration of TP53 gene therapy in small cell lung cancer using nonviral polymeric nanoparticles [15].

Nanoparicles are potential effective carriers with traditional drugs to improve their specificity and efficiency in lung diseases. However, a number of factors can influence the effects of nanoparticles in the lung, such as physical characteristics and toxicity, routes of administration, and lung physiology in the presence of respiratory diseases [23]. Especially, some nanoparticles are found to have adverse effects in animal models. Hence we should be prudent to apply them on treatment of lung diseases in humans.

## 3. The Pulmonary Diseases Caused by Nanoparticle Exposure

With the advent of a large amount of nanoproducts into our life, nanoparticles have existed in our environment, especially in special working places. Inhalation is the primary route by which nanoparticles enter into human bodies. It is reported that some nanoparticles with certain sizes and properties can reach the alveoli areas and deposit in the lung of animal models [24]. The smaller the aerodynamic diameter of the nanoparticles is, the deeper they can travel into the lung. Particles which are smaller than 2.5 micron will even reach the alveoli [25]. Though the mass concentration of inhaled nanoparticles is not high, their tiny diameter, increased surface area and large quantities provide the possibilities that nanoparticles bring the lung irreversible damages [25]. For example, male CD1 mice were subjected to inhaled exposure of CeO_2_ nanoparticles for 28 days at an aerosol concentration of 2 mg/m^3^ in a study [26]. After 14 or 28 days of recovery time, these inhaled CeO_2_ NPs were still located all over the pulmonary parenchyma and a significant bio-accumulation of these particles was observed in the pulmonary and extra-pulmonary tissues, even after one month of post-inhalation exposure [26]. They also observed that a severe, chronic, active inflammatory response including necrosis, proteinosis, fibrosis and well-formed discrete granulomas existed in the pulmonary tissue and tubular degeneration leading to coagulative necrosis occurred in kidneys after inhalation exposure of these nanoparticles. And there have been many works implying that nanoparticles through inhalation or other routes would accumulate in the lung [25]. These works suggest that nanoparticles exposure at low dose may impair the lung tissue and furthermore in extra-pulmonary tissues. Human population studies also indicate that ultrafine particles in the air are risky factor to lead to pulmonary disease and the harmness is correlated with sizes of nanoparticles. Hence, effects of nanoparticles on respiration systems cannot be ignored.

### 3.1. Deposition and Clearance of Nanoparticle in the Lung

The pathogenic effects of inhaled solid materials, including nanoparticles, depend mainly on achieving a sufficient lung burden [27]. The lung burden is determined by the rates of deposition and clearance if solid materials do not interfere with the clearance mechanisms. The physicochemical properties of the material itself are important insofar as they influence deposition and clearance rates [27]. A subchronic 3 months inhalation exposure of rats to ultrafine (~20 nm) and fine (~250 nm) titanium dioxide (TiO_2_) particles demonstrated that the ultrafine particles cleared significantly slower, showed more translocation to interstitial sites and to regional lymph nodes when compared to the fine TiO_2_ particles [28]. Upon nanoparticle penetration into the lung, clearance from the deep lung (alveoli) is predominantly by macrophage phagocytosis. Laboratory exposure studies have shown that if inhaled concentrations are low, which means the deposition rate of the inhaled particles is less than alveolar clearance rate in the lung, then the retention half time is about 70 days (steady-state lung burden during continuous exposure) [27]. On the contrary, if the deposition rate of the inhaled particles exceeds the clearance rate, the retention half time is significantly increased. When inhaled nanoparticles remain in the lung, they may impair or prolong alveolar macrophage-mediated clearance function continuously and interact with the pulmonary epithelial cells. This may result in subsequent lung injury [27].

### 3.2. Pulmonary Diseases after Nanoparticle Exposure

Nanoparticle inhaled exposure to animals may cause long-term or acute lung injury. These changes eventually lead to some types of lung diseases including asthma, chronic obstructive pulmonary disease (COPD), emphysema and mesothelioma and maybe even lung cancer in animal models [10,29,30]. The respiratory system represents a unique target for the potential toxicity of nanoparticles because in addition to being the portal of entry for inhaled particles, it also receives the entire cardiac output. Even, the injury caused by nanoparticles through other routes may indirectly impair the lung without deposition in the lung.

More important, there is a report that uncovered that seven young female workers (aged 18–47 years), exposed to nanoparticles for 5–13 months, all with shortness of breath and pleural effusions were admitted to hospital [31]. Polyacrylate aerosol, consisting of nanoparticles, was confirmed in the workplace. Pathological examinations of patients’ lung tissue exhibited nonspecific pulmonary inflammation, fibrosis and foreign-body granulomas of pleura [31]. Using transmission electron microscopy, nanoparticles were observed in the cytoplasm and caryoplasm of pulmonary epithelial and mesothelial cells, but also located in the chest fluid. These cases arouse concern that long-term exposure to some nanoparticles without protective measures may be related to serious damage to human lungs [31].

#### 3.2.1. Asthma

**Figure 2:**
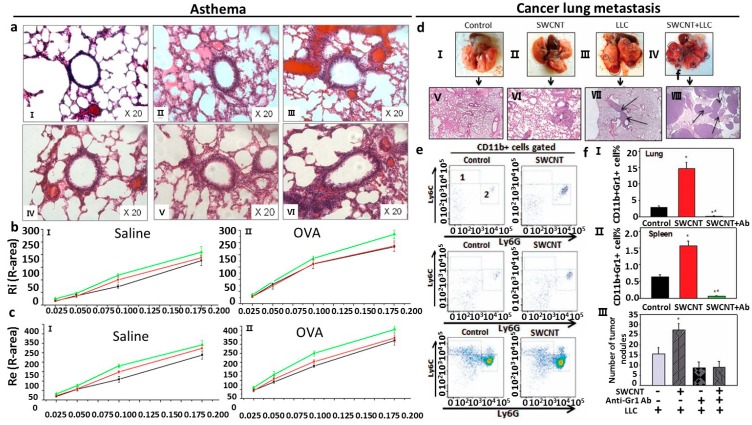
(**a**) Lung section of mice from saline treatment plus 0 mg/mL nano-SiO_2_ exposure (I), saline treatment plus 40 mg/mL nano-SiO_2_ exposure (II), saline treatment plus 80 mg/mL nano-SiO_2_ exposure (III), OVA treatment plus 0 mg/ml nano-SiO_2_ exposure (IV), OVA treatment plus 40 mg/mL nano-SiO_2_ exposure (V), OVA treatment plus 80 mg/mL nano-SiO_2_ exposure (VI) [29]; (**b**) Ri of saline groups (I) and OVA groups (II) [29]; (**c**) Re of saline groups (I) and OVA groups (II) [29]; (**d**) Visualization of the lungs with tumor nodules (cricled) from different groups (I–IV) and histological images of the lung sections (×100) with micrometastases (arrows) from different groups (V–VII) [35]; (**e**) FACS analysis of monocytic CD11b^+^Ly6G^neg^Ly6C^high^ (area 1) and granulocytic CD11b^+^Ly6G^+^Ly6C^l^^ow/neg^ (area 2) MDSC in the lymphoid tissues and lungs in tumor-free mice 48 h after SWCNT (80 μg/mouse) or PBS (control group) pharyngeal aspiration [35]; (**f**) Analysis of CD11b^+^Gr-1^+^ MDSC in tumor-free C57BL6/J mice 48 h after SWCNT (80 μg/mouse) pharyngeal aspiration. * *p* < 0.05 *versus* Control group; ** *p* < 0.0025 *versus* Control group (One-way ANOVA) [35].

As referred above, asthma is a chronic inflammatory response with airway hyperactivity. To study whether pulmonary exposure to nanoparticles causes asthma or asthma-like responses, researchers make use of animal models to observe response [29]. Han *et al.* revealed that after daily intratracheally administered 0.1 mL of 0, 40 and 80 mg/mL nano-SiO_2_ water solutions, respectively to rats for 30 days, nano-SiO_2_ exposure results in adverse changes on aspiratory and expiratory resistance (Ri and Re) in the dose-dependent manner (Figure 2a–c). Lung histological observation revealed obvious airway remodeling in 80 mg/mL nano-SiO_2_-introduced groups. Moreover, nano-SiO_2_ exposure increased the level of IL-4 in the lung [29]. This may be due to the Th1/Th2 cytokine imbalance accelerated by the nano-SiO_2_ through increasing the tissue IL-4 production. This work investigates the relationship between allergic asthma and nano-SiO_2_ for the first time [29]. Additionally, it is reported that a single intratracheal instillation of single-walled carbon nanotubes (SWCNTs) into Institute for Cancer Research (ICR)-mice could induce airway hyperactivity that persisted from 7 days to 6 months after exposure [32]. Transcriptomic analysis showed that SWCNTs might up-regulate proteinases including cathepsin K and matrix metalloproteinase (MMP)12, chemokines C–C motif ligands, and several macrophage receptors. Pathway analyses showed that NF-κB-related inflammatory responses and downstream signals affecting tissue remodeling dominated the pathologic process [32]. The NF-κB inhibitor, pyrrolidine dithiocarbamate, attenuated SWCNT-induced airway hyper-reactivity and chronic airway inflammation [32].

#### 3.2.2. Granuloma

Granuloma is a lesion where kinds of inflammatory cells accumulate and proliferate. Granuloma is substantially considered as chronic inflammation. Granuloma can impair pulmonary functions and is related with other diseases. Pulmonary exposure to some nanoparticles can induce granulomatous response in the lung [33,34]. In an *in vivo* study, spontaneously hypertensive (SH) rats were exposed to PBS or PBS-suspended short or long multi-wall carbon nanotube (MWCNT) particles (0.6 mg/rat) using a non-surgical intratracheal instillation once a day for two consecutive days [33]. It has been shown that after 30 days, SH rats administered long MWCNTs (20–50 μm) but not short MWCNTs (0.5–2 μm) exhibit irreversible granuloma formation in lung tissue [33]. Granuloma formation is one of important markers of lung impair, so the research on the toxicity of nanoparticles in the lung is of great importance.

#### 3.2.3. Lung Cancer

There is no evidence to demonstrate that pulmonary exposure to nanoparticles would induce mesothelioma and even lung cancer (bronchogenic carcinoma). Based on the similarity of some researchers have put forward the hypothesis that pulmonary exposure to nanoparticles may cause carcinogenesis in the lung [10]. Moreover some works have demonstrated that administration of carbon nanotubes by routes other than pulmonary exposure can induce mesothelioma in tissues other than lung [36,37]. Hence, it is possible that nanoparticle exposure can induce mesothelioma in the lung. It should be paid more attention should focus on the possibility that some nanoparticles may promote metastatic growth of tumor cells in animal models. Pathogen-free female C57BL6/J mice received single-wall carbon nanotube (SWCNT) (80 μg/mouse) by pharyngeal aspiration. After 48 h, lewis lung carcinoma cells (LLCs) were injected into these mice via the tail vein and sacrificed 3 weeks later [35]. Exposure of mice to SWCNT prior to tumor cell injection resulted in significant acceleration of tumor growth. SWCNT pre-treatment caused significant, up to 2.5-fold, elevation of the number of visible pulmonary macrometastasis as well as increasing the total area of metastatic nodules upon histopathology evaluation of the lung tissues [35]. The author revealed that carbon nanotubes enhance metastatic growth of lung carcinoma via up-regulation of myeloid-derived suppressor cells (MDSC) (Figure 2d–f). Single aspiration of SWCNT up-regulated accumulation of MDSC in tumor-free and tumor-bearing mice but did not up-regulate immature and regulatory dendritic cell subsets in tumor-bearing mice [35]. And depletion of SWCNT-induced MDSC using Ly-6G/Ly-6C (Gr-1) antibody suppressed metastatic growth of lung tumors [35]. It is implied that inhaled nanoparticle exposure may promote the metastatic growth of cancer cells in patients with certain kinds of cancer and deteriorate their health conditions. The microenvironment, including fibrosis and chronic inflammation, created by inhaled nanoparticles in the lung may facilitate metastatic growth of cancer cells [35].

### 3.3. Pathobiological Processes Caused by Nanoparticles in the Lung

Since nanoparticles can cause severe lung diseases and impair lung function, it is of great importance to understand mechanisms by which these diseases occurred. Four pathobiological processes are considered most relevant to lung injury, including oxidative stress, inflammation, genotoxicity and fibrosis.

**Figure 3:**
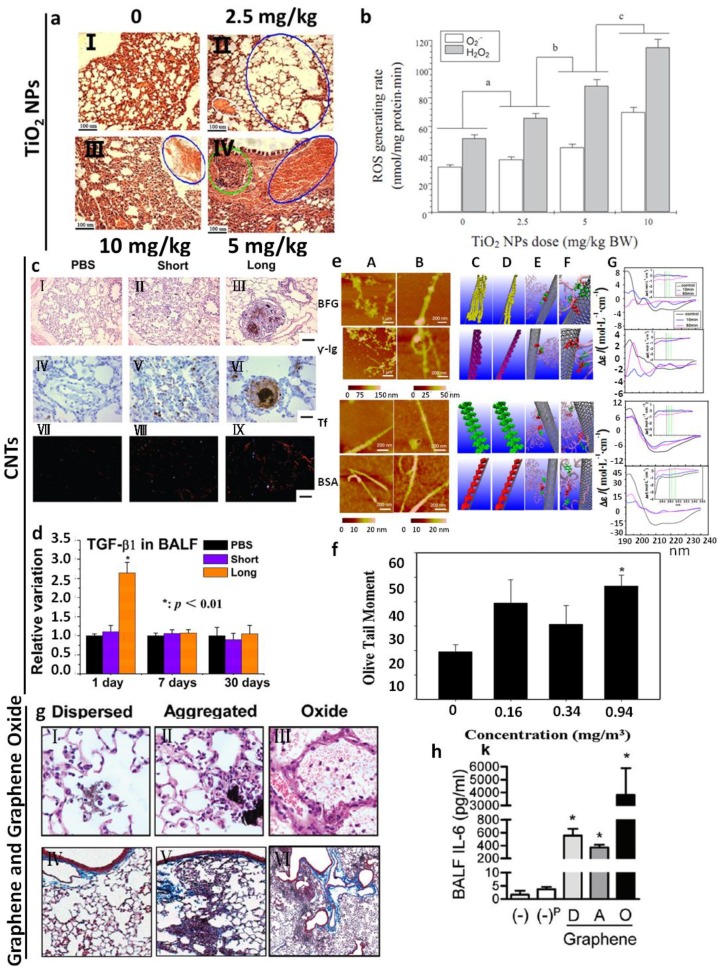
(**a**) Histopathological observation caused by an intratracheal instillation with TiO_2_ NPs for 90 consecutive days from different groups. (I–IV) 2.5 mg/kg TiO_2_ NPs group presents pulmonary emphysema (blue cycle) and edema (white cycle) (II). 10 mg/kg TiO_2_ NPs group indicates pulmonary bleeding (blue cycle) (III). 5 mg/kg TiO_2_ NPs group indicates inflammatory cell infiltration (green cycle) and congestion of blood vessel (blue arrow) (IV) [38]; (**b**) ROS accumulation of the mouse lung after an intratracheal instillation with TiO_2_ NPs for 90 consecutive days. Treatments with different letters indicate significantly different values (*p* < 0.05). Values represent means ± SE (*N* = 5) [38]; (**c**) HE-staining (I–III), immunohistochemistry of FSP-1 (IV–VI) and Sirius Red staining (VII–IX) of lung tissues of SH rats exposed by intratracheal instillation to PBS, short or long MWCNTs for 30 days. Scale bar as follows: 100 μm (I–III, VII–IX) and 50 μm (IV–VI) [33]; (**d**) ELISA analysis of TGF-β1 in BAL fluid of SH rats exposed by intratracheal instillation to PBS, short or long MWCNTs [33]; (**e**) AFM images of Bovine Fibrinogen (BFG), Gamma globulin (Ig), Tf (Transferrin) and BSA (Bovine serum albumin) after incubation with SWCNTs for 10 min (**A**) and 5 h (**B**). Molecular modeling illustrations for proteins (in beads representation) binding to SWCNTs after incubation for 10 min (**C**) and 5 h (**D**). (**E**) Locations of the most preferred binding sites on proteins for SWCNTs. Residues highlighted in van der Waals representation corresponding to tyrosine and phenylalanine. Tyrosine: red, phenylalanine: green, other parts of protein: transparent pink. (**F**) The detailed orientations of aromatic rings of tyrosine and phenylala nine residues interacted to six-member rings of SWCNTs (silver). The tyrosine residues (red) are rendered as licorice representation, and phenylalanine residues (green). (**G**) The far-UV CD spectra of proteins after incubation with SWCNTs and the insets are near-UV CD spectra of proteins incubated with SWCNTs [39]; (**f**) Quantitative analysis of Sprague Dawley rat lung cell DNA damage after whole-body exposure to MWCNTs using comet assay. The Sprague Dawley rats were exposed at 0, 0.16, 0.34, and 0.94 mg/m^3^ for 1 month (* *p* < 0.05) [40]; (**g**) Mice were treated with graphene in 2% Pluronic F 108NF (Dispersed), aggregates of graphene in water (Aggregated) or GO in water by intratracheal instillation and 21 days later, the lungs were examined for markers of fibrosis. Photomicrographs of lung sections from different groups at 200× (I–III) and trichrome stained lung sections (IV–VI) [41]; (**h**) BALF levels of IL-6 from different groups (* *p* < 0.05) [41].

#### 3.3.1. Oxidative Stress

Oxidative stress is caused by an imbalance between the production of reactive oxygen and a biological system’s ability to readily eliminate the reactive intermediates or easily repair the resulting damage. It may be caused directly by generating reactive oxygen species (ROS) in the vicinity or inside the cell or could indirectly affect mitochondrial respiration [42] or deplete antioxidant species within the cell [43]. The severity of the oxidative stress may be an important step in triggering some harmful biological processes [44], like aging. Oxidative stress is one of the most common endpoints reported following the treatment of cells cultured with nanoparticles or respiration exposure to animal models. Here we mainly discuss *in vivo* studies, though *in vitro* studies are also important to understand mechanisms of nanoparticle generating oxidative stress. For instance, after intratracheal instillation of TiO_2_ NPs for 90 consecutive days in mice, TiO_2_ NPs are significantly accumulated in the lung [38]. Exposure to TiO_2_ NPs significantly increased the accumulation of reactive oxygen species and the level of lipid peroxidation, and decreased antioxidant capacity in the lung (Figure 3a,b). Furthermore, TiO_2_ NPs exposure activated NF-κB and increased the levels of cytokines like tumor necrosis factor-α, interleukin-2, interleukin-4, interleukin-6, interleukin-8 and interleukin-10. It is suggested that the generation of pulmonary inflammation caused by TiO_2_ NPs in mice is closely related to oxidative stress and the expression of inflammatory cytokines [38]. In summary, 90-day exposure to TiO_2_ NPs significantly increased the accumulation of ROS and the level of lipid peroxidation, and decreased antioxidant capacity in the lung. ROS generated by NPs and subsquent oxidation of antioxidant species could directly impact the animal health or indirectly trigger other pathobiological processes, like inflammation and genotoxicity [38].

#### 3.3.2. Inflammation

Inhaled nanoparticles, as foreign bodies, are probable to give rise to acute or chronic inflammatory responses, including recruitment of inflammatory cells and release of cytokines [39,41]. In a study, solutions of graphene oxide (GO) were administered directly into the lungs of C57BL/6 mice. 24 h after the administration of GO (50 μg/mouse in a total volume of 50 μL/mouse), researchers observed severe acute lung inflammation with alveolar exudates and hyaline membrane formation [41]. GO was accompanied by a leakage of protein into the alveolar space, broncho alveolar lavage fliud (BAL fluid) pleiocytosis, and elevated BAL levels of pro-inflammatory cytokines [41]. And lung inflammation was apparently observed 21 days after GO administration, however, there was little evidence of lung fibrosis (Figure 3g,h). Also, there is a close connection between oxidative stress in the cell and the elicitation of an inflammatory response via pro-inflammatory gene transcription. Pro-inflammatory pathways such as the mitogen-activated protein (MAP) kinases are oxidative stress-responsive and are activated by some NPs [45]. The redox-responsive NF-κB and activator protein-1 (AP-1) transcription factors have also reported to be activated in NP exposed cells [46,47].

#### 3.3.3. Genotoxicity

A genotoxic substance deleteriously impacts the genome of a cell either by direct or indirect damage to the cellular DNA including effects on the cellular pathways that monitor and protect genome integrity. This could include primary or secondary genotoxicity. Primary genotoxicity is caused by direct binding of the particle with the DNA or component of the cell division machinery such as centromeres or microtubule spindle or intrinsic free radical production [48]. Nanoparticles may cause genotoxicity through both mechanisms. A direct interaction between CNT and DNA has been reported [49]. This implies that CNT may cause primary genotoxicity in cells or *in vivo*. In addition, pulmonary exposure to NPs may cause genotoxicity through the induction of chronic inflammation leading to persistent oxidative stress. In a study, after pulmonary exposure of 0.94 mg/m^3^ MWCNTs to Sprague Dawley rats for 5 days (6 h/day), lung cells were then isolated on day 0 and 1 month after the 5-day exposure, respectively [40]. The animals exhibited no significant body weight changes, abnormal clinical signs, or mortality during the experiment. A single-cell gel electrophoresis assay (Comet assay) was conducted to determine the DNA damage in lung cells obtained from the right lung [40] (Figure 3f). The results demonstrate that MWCNTs caused a statistically significant increase in lung DNA damage at high concentration (0.94 mg/m^3^) when compared with the negative control group on day 0 and 1 month post-exposure [40]. In addition, Gillespie *et al.* demonstrated that nickel hydroxide nanoparticles are capable of inducing inflammatory effects in the lungs after both short- and long-term pulmonary exposure to C57BL/6 mice [50]. Moreover, long-term exposure renders the cell vulnerable to DNA aberrations that consequently lead to mutagenesis [50].

#### 3.3.4. Fibrosis

In a number of studies, fibrosis has been described as an endpoint following inhaled nanoparticle deposition in the lungs. These effects appear to be driven by inflammatory effects, including unusual modes of inflammation including eosinophils [51]. High aspect ratio nanomaterials, especially carbon nanotubes were often reported to cause fibrosis. This may be due to fiber or needle-like shape. In an *in vivo* study, spontaneously hypertensive (SH) rats were exposed to PBS or PBS-suspended short or long multi-wall carbon nanotube (MWCNT) particles (0.6 mg/rat) using a non-surgical intratracheal instillation once a day for two consecutive days [33]. It has been shown that after 30 days, SH rats administered long MWCNTs (20–50 μm) but not short MWCNTs (0.5–2 μm) exhibit increased fibroblast proliferation, collagen deposition and granuloma formation in lung tissue [33] (Figure 3c). Meantime, MWCNTs can significantly activate macrophages and increase the production of TGF-β1, which induces the phosphorylation of Smad2 and then the expression of collagen I/III and extracellular matrix (ECM) protease inhibitors in lung tissues [33] (Figure 3d). Fibrosis was also observed in male C57BL/6 mice 6 weeks after a single inhalation exposure of 30 mg/m^3^ for 6 h [52]. Also, the inhalation of nonpurified SWCNT at 5 mg/m^3^, 5 h/day for 4 days or pharyngeal aspiration (5–20 μg per mouse) causes inflammatory response and oxidative stress culminating in the development of multifocal granulomatous pneumonia and interstitial fibrosis in the lung of C57BL/6 mice [52].

Taken together, these four pathobiological processes above have been often observed at the same time. But there have been still some studies indicated that these processes may not be always positively related [53]. Additionally, there are some types of nanoparticles reported to have no adverse effect on the lung after inhaled exposure to animal models [54]. Physicochemical properties of nanoparticles contribute to the severity of pathobiological processes, like shape, size, length, surface modification and agglomeration. In Table 2, we summarized physicochemical properties of kinds of nanoparticles that may influence the severity of lung injury after exposure. Some modification can indeed reduce adverse effects on the lung [55]. In addition, dose, period and *in vivo* exposure procedures, including whole body, head/nose/mouth-only and lung-only exposures are also involved. These procedures all have advantage and disadvantages. Moreover, nanoparticle exposure through these different procedures may cause pulmonary injury to the different extent. In a comparison study in mice, SWCNT inhalation elicited a stronger inflammatory response and increased oxidative stress than instillation of an equivalent mass. Although the trends were similar in both exposure models, inhalation of the dry powder was more potent for SWCNTs than instillation of the suspension [52]. In rats the opposite has been observed: inhaled ultrafine TiO_2_ particles (21 nm) led to a decreased pulmonary response compared with a similar dose of instilled particles. These results might be explained by differences between the two methods in particle distribution, dose rate, or clearance [56]. However, another study in rats comparing the two administration routes for TiO_2_ particles gave consistent toxicity data for inhalation and instillation [57]. Except for the factors above, other factors may be also important to influence the severity of pathobiological processes in the lung. For instance, interactions of CNTs with some proteins can enhance their biocompatibility [58] and protein-modified nanotubes are reported to be nontoxic or less toxic than the pristine CNT [39] (Figure 3e).

**Table 2:** Physicochemical properties of inhaled NPs are critial factor to cause pathobiological processes.

Nanoparticles	*In Vivo* Exposure Procedure (Dose, Period, Animal Model)	Physicochemical Properties	Lung Injury and Lung Disease	Ref.
MWCNT	intratracheal instillation once a day for two consecutive days; 0.6 mg/rat; 30 days; SH rat	Length	Long MWCNTs (20–50 μm) but not short MWCNTs (0.5–2 μm) exhibit increased fibroblast proliferation, collagen deposition and granuloma formation in lung tissue.	[33]
	instillation 100 µg/mice; 1, 7, 30, 90, o r 180 days; Male Balb/c mice	Suface modification NT1: none NT2: carboxylic polyacid polymer NT3: polystyrene polybutadiene polymethylacrylate(PMMA) Surface area NT1: 227. 54 m^2^/g NT2: 54.1 m^2^/g NT3: 34 m^2^/g	NT1 and NT2, not NT3, induced inflammatory response and these effects were observed 24 h post-instillation and lasted up to 1 month.	[55]
	intratracheal instillation(single); 2 mg/rat; 3 days; female wistar rats	Thickness (diameter) MWCNT9.4: 9.4 ± 0.3 nm MWCNT70: 70 ± 2 nm	Thin MWCNTs induced an inflammatory lung response when instilled in rats. Conversely, thick MWCNTs appeared to be of low toxicity.	[59]
Graphene	intratracheal instillation (single); 50 μg/mouse; 21 days; C57BL/6 mice	Surface modification (covalent oxidation) aggregation	GO increased the rate of mitochondrial respiration and the generation of ROS, activating inflammation.	[41]
Nickel nanowires	Pharyngeal aspiration; 7 days; 50 mg/mice; female C57BL/6 mice	Length Long: 24 ± 7 µmShort: 4.3 ± 1 µm	Long nanowires led to a moderate inflammatory response and a strong granulomatous response in the peripheral airways, but short ones did not cause these responses.	[60]
Nano-TiO_2_	nose-only exposure for 6 h; 20 mg/m^3^; 16 h; Rats	Agglomeration state: Large agglomerate (LA): >100 nm Small agglomerate (SA): <100 nm Size: 5 nm 10–30 nm 50 nm	5 nm SA particles caused a noted increase in cytotoxic effects, while oxidative damage was less compared to 10–30 and 50 nm SA particles. In SA and LA aerosols, the 10–30 nm TiO_2 _NP induced the most marked pro-inflammatory effects.	[61]
	Intratracheal instillation(single); 1 or 5 mg/kg; 24 h, 1 week, 1 month, and 3 months; Male rats	Surface area: (1) Nanoscale rods Dlong = 92–233 nm Dwide = 20–35 nm 26.5 m^2^/g (2) Nanoscale dots: 5.8–6.1 nm spherical 169.4 m^2^/g	No significant difference in pulmonary inflammation for long-term exposure.	[62]

Additionally, nanoparticles, which are exposed through other route except pulmonary exposure, may also cause pathobiological processes in the lung. For instance, Nounou *et al.* revealed that oral administration of ZnO NPs induced lung injury possibly through oxidative stress, inflammatory response and DNA damage in the lung of rats. In their study, rats were divided into four groups. Groups I and II were treated orally with 40 and 100 mg/kg ZnO NPs for 24 h respectively, while Groups III and IV received daily 40 and 100 mg/kg ZnO NPs orally for 1 week [63]. Oral administration of ZnO NPs induced eosinophilia and lymphocytes infiltration in the lungs of all the four ZnO NPs-treated groups. Lipid peroxidation was significantly higher, while levels of reduced glutathione and catalase activity were lower in all the four groups [63]. Levels of lung TNF-α were significantly higher after 24 h at high dose and after 1 week at both doses. Interleukin-1β and pentraxin-3 levels were significantly increased at 1 week only at both low and high doses. Meanwhile, DNA damages were discovered in all the four groups [63].

## 4. Secondary Diseases after Pulmonary Diseases Caused by Nanoparticle Exposure

### 4.1. Cardiovascular Disease

**Figure 4:**
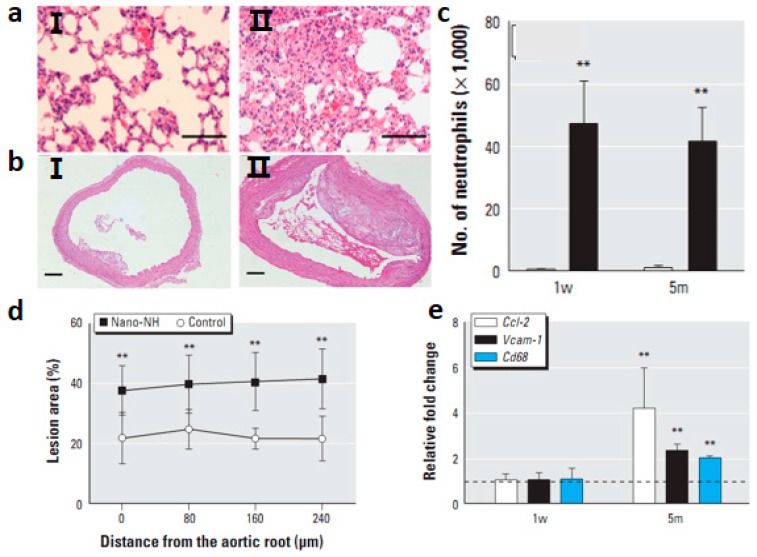
(**a**) HE-stained images from a control mouse (I) and a nano-NH-exposed mouse (II); bars = 0.1 mm [65]; (**b**) Photomicrographs of HE-stained aortic cross-sections from control (I) and nano-NH (II) mice in the 5m (5 month) group; bars = 0.2 mm [65]; (**c**) Number of neutrophils measured in BALF in mice after 1 w or 5m nano-NH exposure (79 μg Ni/m^3^ nano-NH). All markers were measured 24 h after the last exposure; values are mean ± SD (*n* = 6/group). ** *p* < 0.01 compared with control [65]; (**d**) Relative plaque area at four different locations of the ascending aorta (at 80 μm intervals) in the 5m group; values are expressed as mean ± SD (*n* = 7/group). ** *p* < 0.01 compared with control [65]; (**e**) Relative mRNA levels of Ccl-2, Vcam-1, and Cd68 in aortas from the 1 w (1 week) and 5 m (5 months) exposure groups; values are mean ± SD (*n* = 6/group) expressed as relative fold increase over controls (normalized to 1; dashed line). All samples were collected 24 h after the last exposure. ** *p* < 0.01 compared with control [65].

Because there have been reports of associations between inhaled ambient ultra fine particles and increased risk of cardiopulmonary diseases [64], it has been suggested that inhaled NPs might have the potential to induce systemic cardiovascular toxicity as well. Hyperlipidemic, apoprotein E-deficient (ApoE−/−) mice were exposed to nickel hydroxide (nano-NH) nanoparticles at either 0 or 79 μg Ni/m^3^, via a whole-body inhalation system, for 5 h/day, 5 days/week, for either 1 week or 5 months [65]. ApoE−/− mouse was an animal model widely used to study the development of atherosclerosis [65]. Inhaled nano-NH NPs induced significant oxidative stress and inflammation in the pulmonary and extra pulmonary organs, indicated by up-regulated mRNA levels of certain anti-oxidant enzyme and inflammatory cytokine genes (Figure 4a,c). Nano-NH NPs increased mitochondrial DNA damage in the aorta and significant signs of inflammation in BAL fluid [65]. In addition, after 5-month exposures, nano-NH NPs exacerbated the progression of athero sclerosis in ApoE−/− mice (Figure 4b,d,e). Several mechanisms have been proposed to explain how pulmonary NP exposure can elicit cardiovascular responses [65]. Among them, there is a hypothesis proposes that NPs deposited in the lung initiate local inflammatory responses via oxidative stress that further develop into systemic oxidative stress/inflammation [66]. Erdely *et al.* observed that after carbon nanotube deposition in the lung, acute local and systemic responses are activated and characterized by a blood gene and protein expression signature [67]. These studies demonstrated a close cross-talk between the pulmonary and systemic circulation after pulmonary exposure of nanoparticles. These alterations could trigger acceleration of atherosclerosis progression.

### 4.2. Diseases in Other Tissues

Except cardiovascular diseases, inhaled exposure to nanoparticles can induce damages in other tissues. A study demonstrated that cadmium associated with inhaled cadmium oxide nanoparticles impacts fetal and neonatal development and growth [68]. Additionally, a study above demonstrated that these inhaled CeO_2_ nanoparticles were distributed into other tissues, including liver, heart, kidney and even brain and caused tubular degeneration leading to coagulative necrosis in kidneys [26].

It is believed that the damages in these tissues may be mainly due to the direct adverse effects of the nanoparticles deposit through inhalation. Also, it is possible that the inflammatory factors, including cytokines and chemokines, transported through blood stream may impair the other tissues or organs. However, those studies did not clarify whether the damage resulted from or were related to the pathobiological processes in the lung. Hence, it is urgently required for assessments of the relationship between systemic diseases and the pathobiogical processes in the lung.

## 5. Conclusions

**Figure 5:**
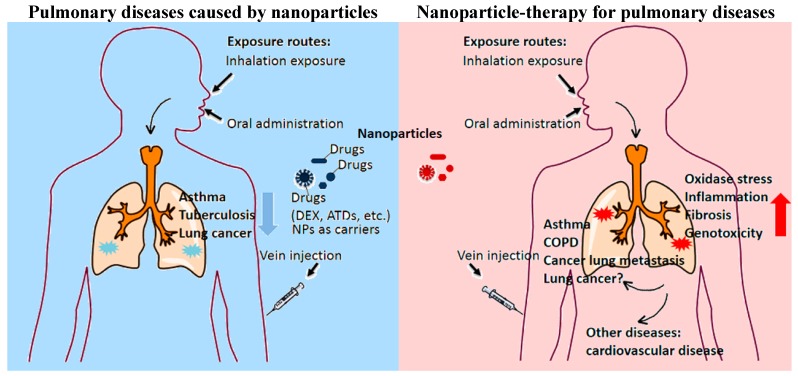
The role of nanoparticles in pulmonary diseases.

Nanoparticles, including engineered nanomaterials, are playing a role as a double-edged sword in lung diseases (Figure 5). Some nanomaterials, designed as drug carriers, would better the curative effect of traditional therapies, while a large amount of nanoparticles are reported to have adverse effects on the lung of animal models and even human bodies after exposure, particularly inhalation exposure. It seems paradoxical, but in fact, would be figured outsome solutions to help solve this paradox.

At first, the design and selection of nanomaterials for medicine use of lung disease are important. Biodegradable polymers are better choices and few are reported to have adverse effects on the lung. Carbon nanotube, metallic nanoparticles and metallic oxide nanoparticles are widely reported to exert adverse effects on the lung. These injuries may partly due to poor biocompatibility of nanoparticles. Some methods are developed to enhance biocompatibility [58]. Second, the development of risk assessment is urgently required. The safety of all engineered nanomaterials and nanoparticles known to be common in our life should be assessed. The recent studies provide us with some evidences and clues to further research on pulmonary impair of nanomaterials. However, many questions remain unclear. First, the physicochemical properties of nanomaterials, exposure routes, exposure doses and periods should be considered to help us compare different studies. Second, nanotoxicological effects considerably depend on nanoparticle size. Since size is not a factor that is considered to lead to toxicological effects of particles at the large scale, new concepts and parameters should be brought into the recent toxicology and new knowledge system should be built. Third, the different properties and states in different media should be discussed, for example, nanoparticle aggregation *in vivo* influenced its original properties.

Next, development of assessing method and standard of the nanoparticle safety is urgent. Current data on nanoparticle toxicity is made use of to assess human’s health hazards, especially professional population’s. Some current reviews have reported health hazard of nanoparticles. Finally, the toxicological studies of nanoparticles should focus on the systemic toxic effects and enhance human population investigation and mechanism study of these harmful effects. Since particles at the nano-scale have different properties, their bio-toxicity may be different from that of particles with sizes greater than micron level. Thus databases and results of safety assessment based on routine substances may not be proper for nanomaterials. Taken together, typical investigations on toxic effects caused by nanoparticle exposure and their mechanisms provide the theoretical basis of safety assessment and standards. These studies will help us to understand the hazardous effects of nanoparticles in the air, especially in industrial waste gas, and ultilize nanomaterials properly.
